# Fecal microbiota transplantation for intractable diarrhea due to severe dysbiosis and cytomegalovirus enteritis: a case report

**DOI:** 10.3389/fnut.2026.1728176

**Published:** 2026-03-30

**Authors:** Xin Zhao, Zheng Lv, Hong Liu, Jiancheng Zhang

**Affiliations:** 1 Department of Critical Care Medicine, Union Hospital, Tongji Medical College, Huazhong University of Science and Technology, Wuhan, China; 2 Key Laboratory of Anesthesiology and Resuscitation (Huazhong University of Science and Technology), Ministry of Education, Wuhan, China; 3 Hubei Jiangxia Laboratory, Wuhan, China; 4 Hubei Key Laboratory of Regenerative Medicine and Multi-disciplinary Translational Research (Huazhong University of Science and Technology), Wuhan, China

**Keywords:** case report, cytomegalovirus enteritis, dysbiosis, fecal microbiota transplantation, multidrug-resistant organisms

## Abstract

**Background:**

Cytomegalovirus (CMV) enteritis can lead to intractable diarrhea, especially when complicated by severe gut dysbiosis, posing a significant therapeutic challenge.

**Methods:**

We present a case of a 40-year-old woman with a history of traumatic brain injury and prolonged broad-spectrum antibiotic use, who developed persistent bloody, mucus-containing diarrhea (up to 40 episodes daily). Colonoscopy with biopsy confirmed CMV enteritis, and 16S rRNA sequencing revealed severe intestinal dysbiosis. Treatment consisted of intravenous ganciclovir combined with multiple sessions of fecal microbiota transplantation (FMT) delivered via jejunal tube, alongside tailored nutritional support.

**Results:**

Despite initial persistence of symptoms, the combined antiviral and FMT regimen led to resolution of diarrhea, normalization of inflammatory markers, and restoration of enteral tolerance. Follow-up colonoscopy showed mucosal healing and negative CMV staining. Microbiota analysis demonstrated restoration of diversity and a shift toward donor-like taxonomic profiles.

**Conclusion:**

This case highlights CMV as an emerging cause of severe enteritis in non-immunosuppressed surgical patients and supports the efficacy of combined antiviral therapy and FMT for refractory diarrhea associated with dysbiosis and CMV infection.

## 1 Introduction

Patients after brain trauma surgery often receive prolonged broad-spectrum antibiotics and remain in a postsurgical stress state, predisposing them to multidrug-resistant infections and intestinal dysbiosis. This cascade can lead to antibiotic-associated diarrhea and may progress to opportunistic enteritis. While cytomegalovirus (CMV) enteritis typically affects immunosuppressed individuals, growing evidence shows it can also cause severe diarrhea in non-classically immunosuppressed postoperative patients. CMV infection may further synergize with gut microbiota disruption to worsen mucosal barrier injury (1, 2). Conventional anti-infective and immunomodulatory therapies often yield poor outcomes in complex enteritis, especially when multidrug-resistant organism (MDRO) colonization is present. Although fecal microbiota transplantation (FMT) is effective for recurrent *Clostridioides difficile* infection (CDI) (3), its role in concurrent CMV enteritis and gut microbiota dysbiosis remains unclear.

This case describes a patient who developed refractory diarrhea after brain trauma surgery and prolonged broad-spectrum antibiotic use, diagnosed with CMV enteritis and gut microbiota dysbiosis. Sequential antiviral therapy and multiple FMT sessions restored the intestinal mucosal barrier and reestablished microbial balance. The case confirms that CMV can trigger enteritis in non-classically immunosuppressed patients and demonstrates a successful treatment strategy for postoperative complex infectious diarrhea. It highlights the essential role of gut microecological regulation amid rising antimicrobial resistance.

## 2 Case presentation

A 40-year-old woman (150 cm, 45 kg) was admitted on July 24, 2025, with 17 days of intermittent diarrhea and fever, worsening over the prior 5 days with bloody, mucus-containing stools. She had been hospitalized since July 6 for recovery from traumatic brain injury when she developed diarrhea (3–4 times daily, yellow watery stools), low-grade fever (up to 38 °C), and vomiting. On July 18, diarrhea increased to up to 40 times daily (about 50 mL each), accompanied by bloody stools, paroxysmal lower abdominal pain, nausea, vomiting (gastric contents and white foam), and palpitations; no chills or persistent fever occurred. Local hospital treatment with liver protection, anti-infectives, and mesalazine failed. She was transferred on July 22 due to clinical deterioration. *Clostridium difficile* toxin gene test was negative. Blood tests showed WBC 14.85 × 10^9^/L, neutrophils 10.43 × 10^9^/L. Stool analysis revealed red blood cells, elevated white blood cells (3+/HP), and phagocytes (2+/HP), no fungi or parasites—indicative of intestinal inflammation. Diagnosed with intestinal infection, diarrhea, malnutrition, and post-brain trauma status, she was admitted to intensive care unit (ICU). Since onset, she had poor mental status, appetite, and sleep; reduced strength; weight loss of 15 kg; urine output remained normal.

Four months earlier, she developed impaired consciousness and speech disturbances after traumatic brain injury, with irrelevant responses. On March 25, 2025, she underwent intracranial hematoma evacuation, decompressive craniectomy, and intracranial pressure monitor placement. A tracheotomy was performed on March 27 for pulmonary infection. On April 30, a left ventriculoperitoneal shunt was placed for hydrocephalus. On May 16, CSF culture identified Gram-positive cocci, confirming intracranial infection; the shunt was removed and vancomycin (44-day course) and meropenem (39-day course) were initiated.

At admission, she was alert but had deficits in calculation, orientation, and cognition, with occasional irrelevant responses. She appeared underweight and was uncooperative. Nasal oxygen was given at 3 L/min; temperature 36.6 °C. Vital signs: HR 95 bpm, RR 15 breaths/min, BP 120/87 mmHg, SpO₂ 100%. No jaundice or lymphadenopathy. A well-healed arc-shaped scar was present in the right frontal-temporal region with bone window depression. Pupils were equal, round, about 2 mm, light-reactive. Neck was supple. Breath sounds were coarse bilaterally, without rales. Cardiac rhythm was regular, no murmurs. Abdomen was flat, soft, non-tender; no edema. Neurological exam: muscle strength grade IV in right upper limb and left lower limb, approximately grade II in left upper limb. Bilateral pathological reflexes were absent.

**Figure 1:**
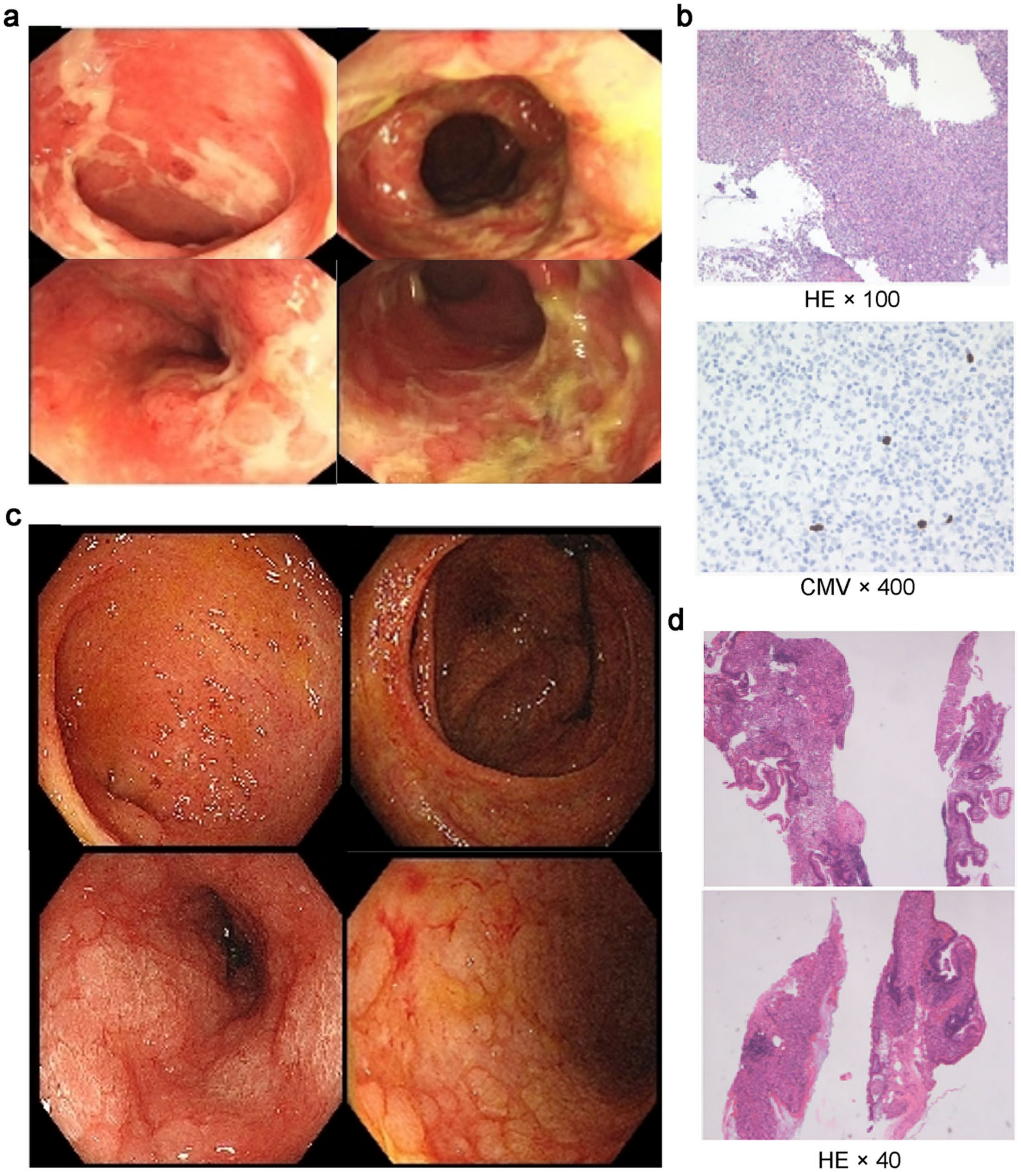
**(a)** Endoscopic examination reveals hyperemia and edema of the terminal ileal mucosa, accompanied by white exudates. Diffuse mucosal inflammation characterized by nodular elevations and fibrinopurulent exudates extends from the ileocecal junction through the ascending, transverse, descending, and sigmoid colon to the rectum. **(b)** Immunohistochemical staining demonstrates focal and scattered cytomegalovirus (CMV)-positive cells, with *in situ* hybridization showing sporadic CMV positivity. **(c)** Follow-up endoscopy shows residual hyperemia in the terminal ileum along with a few small, round ulcers (3–4 mm) without exudate. The colonic mucosa from the ascending colon to the rectum displays persistent hyperemia and edema, with abundant viscous secretions. Scattered shallow ulcers (2–4 mm) are observed in the ascending colon, and the mucosa is diffusely friable. **(d)** Immunohistochemical analysis indicates no detectable CMV expression, and in situ hybridization for Epstein–Barr virus-encoded RNA (EBER) is negative.

**Figure 2:**
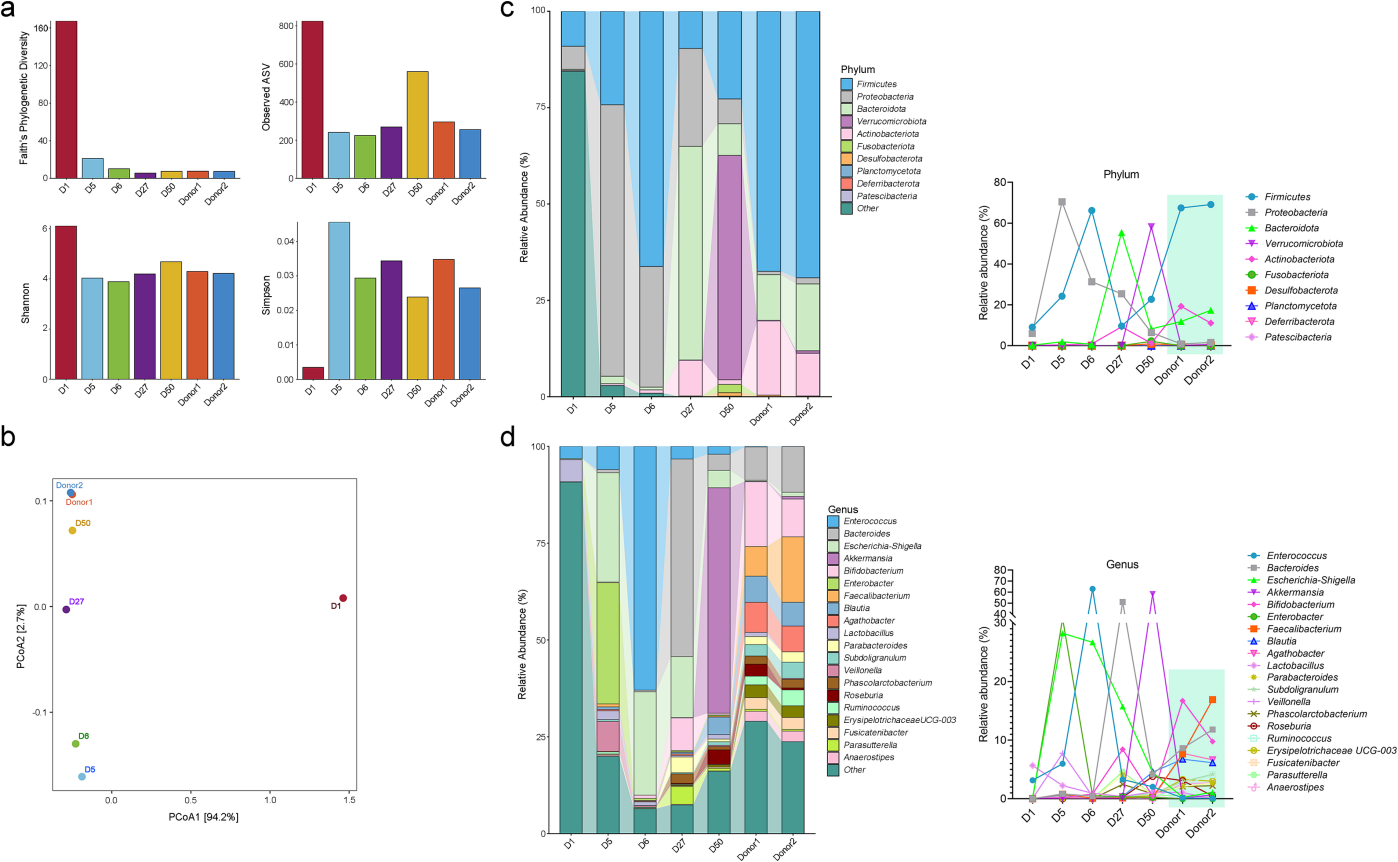
**(a)** Alpha-diversity of the gut microbiome, assessed using Faith’s phylogenetic diversity, observed ASVs, Shannon index, and Simpson index. **(b)** Weighted principal coordinates analysis (PCoA). **(c)** Stacked bar charts illustrating the taxonomic composition of the gut microbiota at the levels of phylum **(c)** and genus **(d)**, with each taxon represented by a distinct color.

After admission, stool analysis showed abundant white blood cells (++++). Tests for *C. difficile* toxin gene, *Salmonella*, and *Shigella* were negative. Autoimmune serology (ENA, ANCA 4-panel + GBM) was negative; serum IgE normal. *Coxsackievirus* B3/B5 IgM, enterovirus RNA, and CMV IgM were negative. Plasma EBV-DNA undetectable; CMV-DNA < 4 × 10^2^ copies/mL. On D6 (July 29), abdominal CT showed mild bowel wall thickening, edema, and irregularity in the colon and distal small intestine, with intraluminal fluid and no obstruction. Colonoscopy on D7 (July 30) revealed hyperemic, edematous terminal ileal mucosa with adherent white exudate; diffuse inflammation from ileocecal junction to rectum with hyperemia, edema, nodular elevations, and dirty coating (Figure 1a). Rectal biopsy showed ulceration with granulation tissue and chronic inflammation, no granulomas. Immunohistochemistry showed focal CMV-positive cells; CD3 (focally positive), CD20 (−), CD56 (−); EBER (−), CMV (sparsely positive); PAS (unremarkable), acid-fast stain (−) (Figure 1b). Diagnosis: CMV-associated infectious colitis. Analysis of intestinal microbiota alterations by 16S ribosomal RNA (rRNA) gene sequencing revealed severe dysbiosis in the patient (Figure 2).

FMT was initiated on D2 (July 25). Prior to the first FMT, the patient received vancomycin 500 mg q12h via nasogastric tube for 48–72 h to reduce intestinal pathogenic load. FMT was performed daily between 11:00 and 13:00 through a nasojejunal tube, using 50 mL of fecal microbiota suspension (provided by Meiyitian Biomedical (Wuhan) Co., Ltd.) per session. Each 50 mL dose contained no less than 3.33 × 10^13^ viable microbial cells, and all donor suspensions underwent 16S rRNA sequencing to ensure safety and compositional consistency. FMT was administered when the patient’s body temperature was below 38.5 °C, and the suspension was pre-warmed to 37 °C before infusion. Despite this, diarrhea persisted. Intravenous ganciclovir 0.25 g q12h was started on D7. FMT continued daily until D10 (August 2). On D6, commercial enteral formula triggered fever and abdominal pain; it was stopped. She was switched to minimal oral intake (<100 mL/day white rice porridge) with full parenteral nutrition. By D11 (August 3), stool leukocytosis resolved. A customized enteral nutrition plan was initiated via nasogastric tube, with rate and volume adjusted per tolerance scoring.

On D15 (August 7), she developed high fever (39.4 °C). Repeat chest CT showed new pulmonary infiltrates, indicating acute lung infection. Empirical therapy: ganciclovir 0.25 g intravenous (i.v.) q12h, cefoperazone-sulbactam 3.0 g i.v. q12h, ornidazole 0.5 g i.v. q12h. On D17 (August 9), sputum culture grew *Enterobacter cloacae* and *Staphylococcus aureus*; urine culture grew *Escherichia coli*. Linezolid 0.6 g i.v. q12h was added. Fever resolved and C-reactive protein (CRP) declined steadily. On D19 (August 11), repeat colonoscopy showed hyperemic terminal ileal mucosa with small round ulcers (3–4 mm, clean bases); ileocecal region largely intact. Ascending colon to rectum showed diffuse congestion, edema, viscous secretions. Scattered superficial ulcers (2–4 mm) in ascending colon; mucosa friable (Figure 1c). Histopathology: (1) Terminal ileum—villous atrophy, mixed lamina propria inflammation, focal cryptitis, apoptotic bodies, localized ulceration; no granulomas or CMV inclusion bodies; CMV IHC and EBER ISH negative; (2) Ileocecal and rectal biopsies—preserved crypt architecture with occasional distortion; focal chronic inflammation, rare apoptotic bodies; (3) Ascending and descending colon—crypt atrophy, reduced goblet cells, diffuse inflammation, small granulation foci. Findings consistent with infectious enterocolitis (Figure 1d). On D20 (August 12), metagenomic next-generation sequencing (mNGS) of intestinal tissue detected 14 reads of CMV and 36 reads of *Candida parapsilosis*. Antimicrobial regimen was adjusted: cefoperazone-sulbactam and ornidazole discontinued; ganciclovir 0.25 g i.v. q12h, linezolid 0.6 g i.v. q12h, and caspofungin 0.5 g daily continued.

The patient remained afebrile. On D21 (August 13), inflammatory markers showed CRP 2.98 mg/L, WBC 7.54 × 10^9^/L, and neutrophil percentage 62.8%, indicating effective infection control. Given clinical stability, linezolid was discontinued; intravenous ganciclovir 0.25 g every 12 h and caspofungin 0.05 g daily were continued for ongoing antiviral and antifungal therapy. From D22 (August 14) to D27 (August 19), FMT was administered daily via jejunal tube with 25 mL of microbial preparation. Stool output gradually decreased and consistency improved; on D25 (August 17), daily volume was 780 mL, yellow and loose with small amounts of semi-solid material. Enteral nutrition was well tolerated, and feeding volume was progressively increased. By D28 (August 20), total enteral intake reached 900 kcal/day.

Repeated FMT significantly reduced severe microbial dysbiosis, as reflected by improvements in both α-diversity and β-diversity metrics (Figures 2a,b). At the phylum level, multiple FMT treatments induced a shift in the relative abundances of key bacterial taxa—specifically *Firmicutes*, *Proteobacteria*, and *Bacteroidota*—toward donor-like configurations. At the genus level, the compositional profiles of several clinically relevant genera, including *Enterococcus*, *Bacteroides*, *Escherichia-Shigella*, *Enterobacter*, *Blautia*, *Lactobacillus*, and *Parasutterella*, were progressively restored (Figures 2c,d).

**Figure 3:**
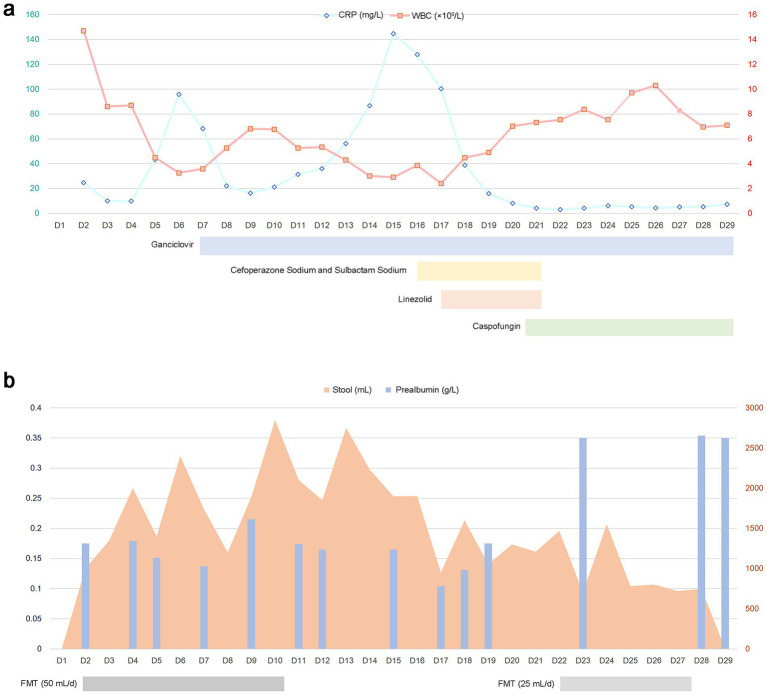
**(a)** Temporal trends in inflammatory markers, including C-reactive protein (CRP) and white blood cell (WBC) count, along with concomitant antibiotic administration. **(b)** Clinical course depicting the fecal microbiota transplantation (FMT) regimen, daily stool volume, and serial prealbumin levels over time.

On D29 (August 21), the patient was transferred to a rehabilitation facility (Figure 3a). After systematic treatment and close monitoring, symptoms markedly improved, stool frequency declined, and enteral tolerance increased, leading to sustained stability and transition into recovery (Figure 3b). At follow-up on October 20, she had fully resumed oral intake, with 1–2 yellow, pasty bowel movements daily and no gastrointestinal symptoms such as diarrhea or abdominal pain.

## 3 Discussion

CMV enteritis is typically an opportunistic infection in immunocompromised individuals, including those with HIV/AIDS, cancer, organ transplants, or on immunosuppressive therapy (4–9). However, rare cases occur in immunocompetent hosts, where CMV can cause severe, refractory diarrhea (10–12). Diagnosis is confirmed by histopathology showing viral inclusion bodies or positive CMV staining (13). In immunosuppressed states, CMV may reactivate locally in the gut without systemic viremia (14). In this case, postoperative CMV infection likely resulted from physiological stress following traumatic brain injury (2).

Dysbiosis played a central role. It involves gut microbiota imbalance—reduced diversity, loss of butyrate-producing bacteria, pathogen overgrowth, and impaired short-chain fatty acid (SCFA) production—driven by broad-spectrum antibiotics and poor diet (15). Antibiotics reduce microbial richness, alter composition, and disrupt metabolic profiles (16), promoting dysbiosis and damaging the intestinal barrier via weakened mucus and tight junctions (1). Dysbiosis is a hallmark of recurrent CDI, IBS, and IBD, contributing to barrier dysfunction and metabolic disturbances. FMT restores microbial balance, SCFA production, and gut barrier integrity; modulates serotonin pathways; and inhibits pathogenic biofilms (17). After FMT, the patient showed increased beneficial taxa (e.g., *Firmicutes*, *Bacteroidota, Lactobacillus, Blautia, Parasutterella*) and reduced pathogens (e.g., *Escherichia-Shigella*, *Enterococcus*)—a key mechanism in treating antibiotic-associated diarrhea (AAD). FMT alleviates bloating, pain, and diarrhea, supporting recovery by reshaping the gut environment (18). Kusakabe et al. reported resolution of Crohn’s-like lesions and CMV enteritis after one FMT in a transplant patient unresponsive to standard therapy, linking mucosal healing to restored microbial diversity following *Enterococcus* overgrowth (19).

Reported clearance rates of MDRO colonization after FMT range from 37.5 to 87.5%, highlighting its potential in combating antimicrobial resistance by inhibiting resistance gene transfer, restoring barrier function, and suppressing pathogen engraftment (20). Antibiotic use reduces microbial diversity and alters taxonomic abundance, fostering MDRO emergence (21, 22). Crum-Cianflone et al. documented successful eradication of multiple MDROs with FMT in a recurrent CDI patient (23).

In adults with recurrent CDI and intact immunity, FMT achieves significantly higher cure rates than antibiotics, reaching 95.56% (3, 24). FMT is now a core therapy for dysbiosis-related conditions through microbiota modulation (25). Rescue FMT is a vital salvage option for critically ill ICU patients with AAD or CDI who fail conventional treatment, especially when complicated by multidrug-resistant infections (26).

This case highlights the dynamic clinical course and impact of timely diagnosis and intervention. Initial symptoms mimicked bacterial infection, but CMV enteritis was ultimately diagnosed, emphasizing the need for early recognition in complex diarrheal diseases. Intestinal dysbiosis significantly contributes to severe diarrhea (27). A combined strategy of prompt antiviral therapy and restoration of microbial homeostasis may offer a more effective, comprehensive treatment approach.

Despite the encouraging outcomes, this study has several limitations. First, as a single case report, the findings are inherently limited in generalizability to broader populations. Second, the absence of a control group precludes direct comparison with alternative treatment strategies or the natural disease course. Third, it is difficult to definitively attribute the clinical improvement solely to the combination of ganciclovir and FMT, as the patient also received concomitant antibiotics and nutritional support. Nonetheless, the temporal association between FMT administration and clinical improvement, along with the restoration of gut microbiota diversity, supports its potential role in recovery. Fourth, the follow-up period was relatively short, and long-term outcomes, including the risk of recurrence, remain unknown. Finally, potential confounders such as the patient’s underlying critical illness, prior antibiotic exposure, and nutritional status may have independently influenced gut microbiota recovery and clinical response. These limitations should be considered when interpreting the results, and future studies with larger cohorts and controlled designs are warranted to validate our findings.

## Data Availability

The 16s rRNA data presented in the study are deposited in the NCBI Sequence Read Archive (SRA) repository, accession number PRJNA1441808.
